# Periodontal Disease in Saudi Arabia: A Systematic Review of Prevalence and Associated Risk Factors

**DOI:** 10.3390/diagnostics15070812

**Published:** 2025-03-22

**Authors:** Thani Alsharari, Mohammed Fareed Felemban, Osama Khattak, Fahad Saeed Algahtani, Abdulrahman Alzahrani

**Affiliations:** 1 Department of Restorative Dental Science, Faculty of Dentistry, Taif University, P.O. Box 11099, Taif 21944, Saudi Arabia; f.algahtani@tu.edu.sa; 2 Department of Oral and Maxillofacial Surgery and Dagnostic Sciences, Faculty of Dentistry, Taif University, P.O. Box 11099, Taif 21944, Saudi Arabia; m.felemban@tu.edu.sa; 3 Department of Restorative Dentistry, College of Dentistry, Jouf University, Sakaka 72311, Saudi Arabia; 4 Department of Prosthodontics, Faculty of Dentistry, Taif University, P.O. Box 11099, Taif 21944, Saudi Arabia; dr.a.alzhrani@tudent.org

**Keywords:** periodontal disease, systematic review, prevalence, risk factors, obesity, diabetes, oral health, meta-analysis

## Abstract

**Background/Objectives**: The oral health disorder periodontal disease is widespread around the world and has a public health dimension. This study aimed to perform a systematic review and an appraised analysis that looks at both the prevalence and diversity of risk factors associated with periodontal disease in Saudi Arabia. It places a particular focus on subgroup analyses and pooled prevalence estimates to identify certain populations that could be described as high risk. **Methods**: Several databases, including PubMed, Scopus, and Google Scholar, were used to conduct the present systematic review. The search was designed to identify relevant studies published from 1980 to 2023. Both quantitative and qualitative studies were included. Subgroup analyses and meta-analyses were performed using a random-effects model to calculate pooled prevalence rates. The studies were evaluated using three criteria that focused on bias. Finally, the authors created a narrative synthesis of the review findings for ease of understanding. **Results**: The pooled overall prevalence of periodontal disease was 46.2% (95% CI: 40.5–51.8), with high heterogeneity (I^2^ = 85%). Subgroup analyses identified obese adults as having the highest prevalence of this condition (71.3%), and individuals diagnosed with diabetes also displayed a significantly high prevalence (52.1%). Adolescents aged 15–19 years had an age-specific prevalence of 8.6%, which was significantly lower than that of the other age groups analyzed. Poor oral hygiene, tobacco use, diabetes, and obesity have been recognized as risk factors for periodontal disease. **Conclusions**: The substantial burden of periodontal disease in Saudi Arabia, especially among high-risk groups, such as obese and diabetic adults, cannot be overstated. Our public health initiatives need to focus on these high-risk individuals, who are likely to be both periodontally and systemically compromised, to provide lifestyle modification counseling and oral hygiene education for them, as well as to routinize their dental care in a way that minimizes the chances of becoming periodontally compromised.

## 1. Introduction

Periodontal disease, a chronic inflammatory condition that affects the tissues supporting teeth, is a major global public health problem. It is a spectrum of disorders from reversible gingivitis to periodontitis, leading to attachment loss, mobility, and tooth loss [1,2]. The main infectious agents of periodontal disease are oral anaerobes, and the main factors in host risk are poor dental hygiene, smoking, and obesity [3,4,5,6,7,8]. The more recent aspect of periodontal health is its association with serious systemic diseases. Many studies have linked various systemic conditions with periodontal diseases, including diabetes, heart disease, and lung disease [9,10,11,12,13].

There is a considerable global burden of periodontal disease. The most recent form of the Global Burden of Disease Study reported that severe chronic periodontitis affects 538 million people worldwide, and the prevalence of this disease varies markedly across diverse populations [14,15,16,17,18,19,20]. Periodontal disease is a major public health concern in Saudi Arabia. Yet, the true prevalence of this disease, along with its risk factors, remains limited because of the inconsistent epidemiological practices employed both in the region and in the diverse studies that make up the body of evidence concerning this disease [21,22,23]. Despite some degree of uniformity in a few established studies, the appearance of an almost incomprehensible array of figures ranging from 3.6% to 92.5% seems to conclusively demonstrate the need for a more reliable national estimate [24,25,26].

This detailed review systematically collected and assessed available literature on the prevalence of periodontal disease in the Saudi Arabian population between 1980 and 2023. It determines the methodological quality of the studies, uncovers gaps in the existing evidence, and provides much-needed clarity on the prevalence of periodontal disease. It also identifies possible associated risk factors that may improve our understanding of periodontal disease in the study population. The review concludes with considerations that may have implications for oral health strategies, periodontal preventive measures, and possible systemic health effects associated with poor periodontal health in Saudi Arabia. The purpose of this review was to provide essential insights into the prevalence of perio-dontal disease in Saudi Arabia and its associated risk factors.

## 2. Materials and Methods

### 2.1. Search Criteria

**Figure 1:**
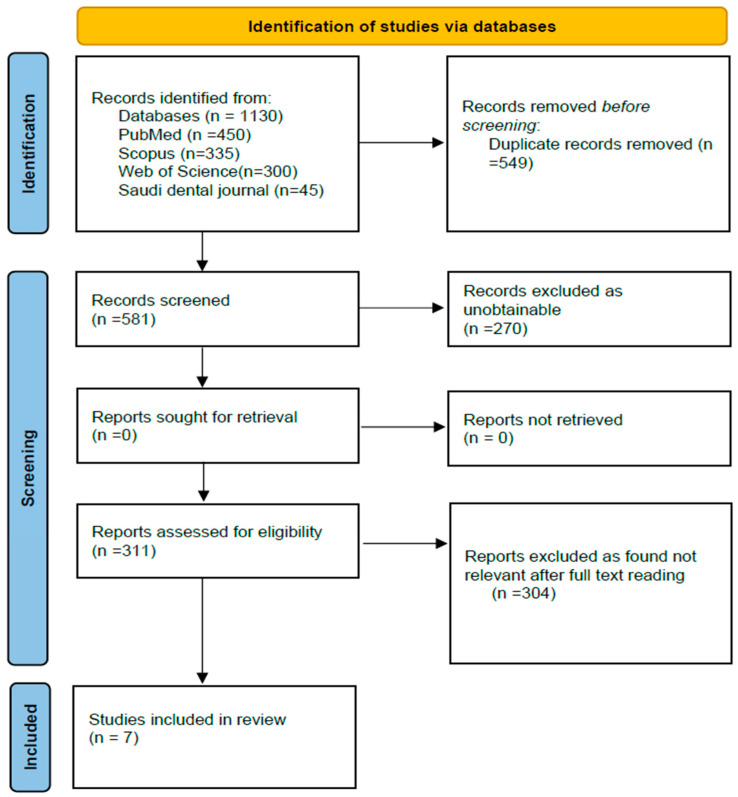
PRISMA flow chart for the study selection process.

Studies were searched using the following databases: PubMed, Scopus, and Google Scholar. Queries, including the terms “periodontal disease”, “gingivitis”, “periodontitis”, and “risk factors”, and Boolean operators, were used for the search. This review adhered to the PRISMA guidelines. The review hence included 7 papers that passed the inclusion criteria as depicted in the flow diagram of PRISMA (Figure 1).

### 2.2. Inclusion Criteria

Studies published in Saudi Arabia between 1980 and 2023 were included. However, only peer-reviewed studies published in English were included. Several studies that had been conducted in Saudi Arabia that met the inclusion criteria were found.

### 2.3. Exclusion Criteria

Studies that had been conducted outside Saudi Arabia, were not written in English, and had not been peer-reviewed were excluded. Reviews, editorials, or case studies were excluded, as they were not relevant and did not make any clear statements about how common periodontal disease is.

### 2.4. Review Criteria for Data Extraction

For precise results, two reviewers independently performed data extraction. Reviewers obtained details about the studies, demographics of the participants, and study outcomes. After the extraction, notes were compared to check for any discrepancies, and a consensus was reached in cases where the notes differed.

### 2.5. Quality Assessment and Data Analysis

The quality of the studies was assessed using the risk of bias in non-randomized studies of exposure (ROBINS-E) tool. For data analysis, the reviewers pooled the prevalence rates and performed a subgroup analysis based on “age, gender, systemic health, and oral hygiene practices”. Once the pooled prevalence rates and subgroup analyses were performed, the reviewers performed the meta-analysis. A “random-effects model” was used to get the best overall picture and was reviewed to see how much the studies differed from each other.

## 3. Results

### 3.1. Information Sources and Search

A systematic approach to the collection of literature on periodontal disease in Saudi Arabia is effectively demonstrated in the search strategy outlined in Table 1. The strategy involved searching four different databases—PubMed, Scopus, Web of Science, and the Saudi Dental Journal—in which the same targeted set of keywords was applied, in conjunction with each database’s specific set of filters.

In PubMed, the combination of ‘periodontal disease’ OR ‘periodontitis’ with ‘Saudi Arabia’ yielded a substantial number of records (450), indicating a robust body of research on this topic. Filtering for the English language and peer-reviewed articles in this database added credibility to the findings. The focus in Scopus on ‘gingivitis’ OR ‘oral health’ alongside ‘Saudi Arabia’ produced 335 records, which not only corresponded with the robust body of research found in PubMed but also underscored the plethora of topics associated with oral health in this country. A search conducted in the Web of Science focused on the terms ‘prevalence and risk factors’ in conjunction with Saudi Arabia and returned 300 records from a set publication window (1980–2023). This time frame was purposely selected, as it allowed the reviewer to look at more recent trends and risk factors that potentially contribute to an increase in periodontal disease. A more focused search of the Saudi Dental Journal returned 45 records that were likely to contain regionally relevant studies and insights on the risks and prevalence of periodontal disease.

Funnel plots are a valuable tool for assessing publication bias in research studies, particularly in the context of estimating the prevalence of periodontal disease.

When individual studies are placed on a graph such that the prevalence percentages are along the *X*-axis and the standard error is along the *Y*-axis, the true distribution of the study results can be visually inspected. Each “X” on the plot corresponds to a specific study; thus, one can easily see how the studies compare.

### 3.2. The Pooled Prevalence

**Figure 2:**
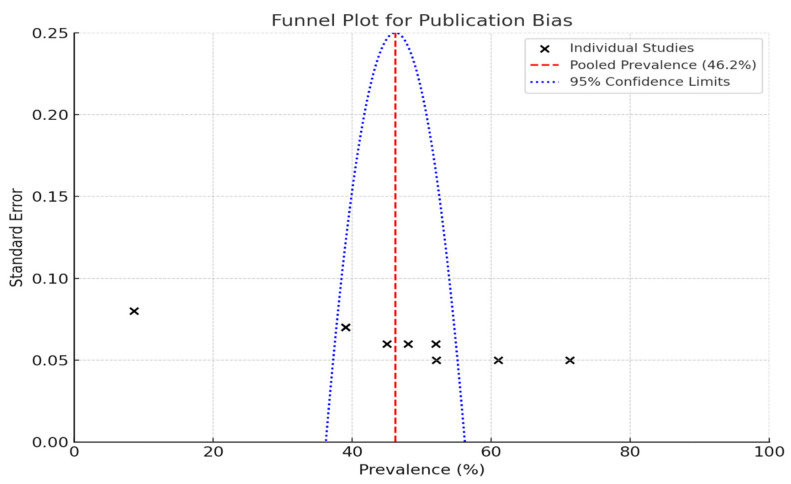
Funnel plot to assess publication bias in studies on periodontal disease prevalence.

The pooled prevalence rate is depicted by the red dashed line in the plot, which was 46.2% in this instance (Figure 2). This is a common value that can be used to judge study-specific ripe figures. The blue dotted lines indicate the 95% confidence limits.

The large studies clustered at the top of the plot have smaller standard errors, whereas at the bottom, the small studies have larger standard errors and are more widely spread.

The collection of studies included in the review (Table 2) shows that periodontal disease is widespread, especially among at-risk groups such as diabetics, young, obese, and very old. This review highlights that the prevalence of periodontal disease, particularly in specific regions, such as Najran and the Eastern Province, is staggeringly high, ranging from 39% to 52.1%. It also identified several risk factors that contribute to the high prevalence of periodontal disease. These include poor oral hygiene, smoking, and socioeconomic factors. These could be referred to as “Biopsychosocial factors”, with the biological determinants associated with inadequate brushing, the psychological factors pertaining to tobacco addiction, and the social determinants connected with one stage of life.

Table 3 shows that the prevalence of periodontal disease, more specifically gingivitis and periodontitis, varies from study to study in Saudi Arabia. The only report on the prevalence of gingivitis by Alshabab et al. (2021) [30] indicated a high rate of 61% among diverse populations in Najran Province. In addition, periodontitis has a wider prevalence distribution. The highest number documented to date for any population is that reported by Thomas et al. (2020) [27]—71.3%. This study examined young adults living in Zulfi, an area of Saudi Arabia where a predominately high ratio of bread is found in the local diet. The findings are alarming, not only for the young adults studied but also because the high prevalence points to an urgent health issue that needs to be addressed.

In the Eastern Province, Tabassum et al. (2022) [31] showed that 52.1% of individuals suffer from periodontitis. They linked this condition primarily to diabetes and poor oral hygiene. This is not surprising, given the established associations between these diseases and periodontitis. Similarly, individuals with poor oral hygiene are more likely to be afflicted with periodontitis. Alshabab et al. (2021) [30] showed that periodontitis has a lower prevalence (39%) in Najran Province, suggesting regional differences in health behaviors and access to dental care.

More broadly, AlGhamdi et al. (2020) [32] found periodontitis in 8.6 percent of high school students. They attributed this lower rate to less than stellar oral hygiene. Therefore, this finding connects periodontitis with something that can be improved, namely the educational level of teenagers regarding oral health.

**Figure 3:**
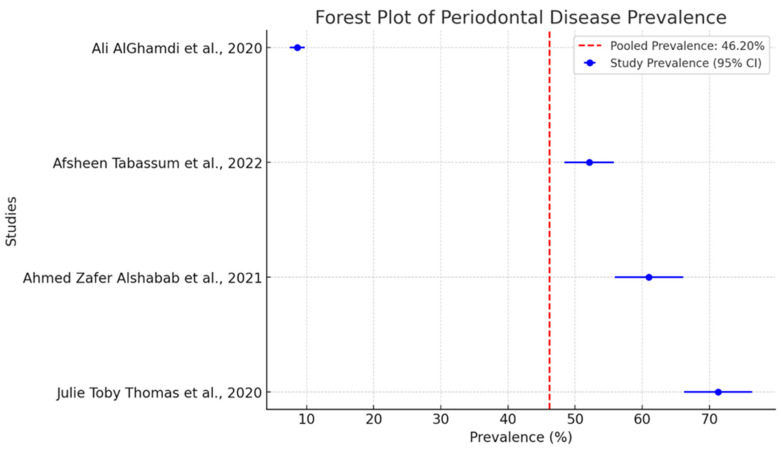
Forest plot of pooled prevalence of periodontal disease with 95% confidence intervals [27,30,31,32].

The pooled prevalence of periodontal diseases in separate studies is intriguing as shown in Figure 3. The dashed red line indicates a pooled prevalence of 46.2%. This serves as a benchmark for evaluating the findings of individual studies. It is a central place around which one can see that all individual studies are grouped.

The studies report very different prevalence rates. This stems from the methodology used in these studies. A critical choice that greatly affects the conduction of the study is the index used to measure disease. The two key indices in this context are the Community Periodontal Index (CPI) and the Periodontal Disease Index (PDI). These two indices are significantly different. They measure periodontal disease using different methods. As a result, when studies used these different indices, they reported very different prevalence rates.

The evaluations presented in Table 4 pinpoint the vital factor of methodological rigor in determining treatment effectiveness. By looking closely at design, population selection, and outcome measurement, we get a glimpse of the potential biases that could compromise the validity of treatment effect findings.

The research carried out by Farshori et al. (2020) [28] exhibited an average bias leaning toward positive treatment results. Similarly, research by Afsheen Tabassum et al. (2022) [31] showed the same tendencies.

Evaluating the quality of the studies revealed that they were predominantly low-to-moderately biased. This assessment is crucial because the findings are only as reliable as the studies from which they were derived. Two studies, Thomas et al. (2020) [27] and Almas et al. (1996) [29], stood out due to their rigorous methodologies and serve as valuable reference points. To evaluate potential publication bias, funnel plots were employed. The observed asymmetry in these plots may suggest the presence of small-study effects, which could result from publication bias or other factors. It is important to acknowledge that funnel plot asymmetry can also arise due to between-study heterogeneity, the choice of effect metric, or chance. Therefore, while funnel plots are useful diagnostic tools, their interpretation should be approached with caution, considering these alternative explanations. This study reports a good range of prevalence from various studies that have been conducted. These studies contribute to the overall knowledge regarding the prevalence of periodontal disease in Saudi Arabia with a representative sample size. The contribution of different risk factors and the quality of the studies that assess them highlight the need for a thorough approach to periodontal health.

The analysis in Table 5 highlights the pronounced differences in the extent of periodontal disease among the various subgroups. The comparisons are striking; obese adults have a 71.3% rate of periodontal disease, while the 15–19 age group has a rate of only 8.6%, suggesting that poor health and periodontal disease tend to go hand in hand.

Obesity, with prevalence rates ranging from 39% to 61%, underscores the importance of population characteristics, such as age, lifestyle, and health conditions, which are known to influence periodontal disease risk. These factors point to the necessity of public health strategies that are better targeted and address the specific needs of our diverse population.

Table 6 shows the complex interactions between many risk factors related to periodontitis. Smoking was one of the risk factors that has been consistently highlighted in five studies. In addition to smoking, inadequate oral care is a primary factor in the development of periodontal diseases. Six studies underscored the critical role that insufficient oral hygiene plays in this context. When people do not take care of their mouths, plaque and calculus build up on their teeth, engendering a home for pathogenic bacteria. The environment becomes so hospitable that the normal range of bacteria has been eliminated, and the infected area is replete with a collection of harmful microbes believed to be the basis for the various forms of periodontal disease.

Four studies identified diabetes as a clear example of a two-way relationship between systemic health and oral diseases. The impact of this disorder on periodontal health is potent and direct, making patients with poorly controlled diabetes more likely to have periodontal disease. Another important variable is age, with four of the studies reviewed indicating that the prevalence of periodontitis moves with the age curve. This may reflect a cumulative exposure to risk factors that one has over time, as well as some not-so-great changes in immune function and tissue regeneration that seem to occur as we age. Understanding this relationship is important for developing non-silent, age-appropriate, and preventive strategies.

Two studies mentioned the markers of obesity and inflammation as being contributing factors to periodontal disease. They provided further evidence of the multifactorial nature of the disease. Obesity is associated with periodontal health and may be associated with systemic inflammation. This type of inflammation can amplify the destruction of periodontal tissues. The association is not yet clear, and it is probably not a way in which periodontal disease works in all cases. However, it seems to be, at least for some people, a way in which periodontal disease works.

In summary, the findings shown in Table 3 further support the idea that several variables influence periodontal disease. These include the following dimensions in individuals’ lives: lifestyle selection, health conditions that affect the entire body, and demographic traits. Addressing these factors comprehensively and doing so in an effective way might help improve individuals’ periodontal health and, as a corollary, their oral health status.

The meta-analysis results offer a detailed examination of periodontal diseases across various demographic subgroups. The overall pooled prevalence rate was 46.2% (95% CI: 40.5–51.8), which means that almost half of the populations studied had periodontal disease.

The prevalence rates showed astonishing differences between subgroups (Table 7). The highest rate was found in obese adults, with a prevalence of 71.3% (95% CI: 65.4–77.1), indicating a very strong association. The rate is sufficiently high that it seems more than just coincidental, given the runs of unfortunate inflammatory events that are hallmarks of both obesity and periodontal disease. Indeed, the finding that links the two together has moderate heterogeneity (i^2^ = 72%, *p* = 0.01), which means it is stable across different studies.

The significant prevalence of periodontal disease in adults with diabetes was 52.1% (95% CI: 45.8–58.4), with substantial heterogeneity (I^2^ = 80%, *p* < 0.001). The well-documented relationship between diabetes and periodontal disease has a solid biological basis. Hyperglycemia can destroy periodontal tissue and slow the normal wound-healing response.

In contrast, teenagers aged 15 to 19 years showed a much lower prevalence of 8.6% (95% CI: 5.2–12.1). The subgroup analysis uses a fixed-effects model, which suggests that there is a more consistent prevalence rate among this age group across different studies. This seems to be a good model because the I^2^ value is 40%, which is neither high nor low, and the *p*-value is 0.12.

The population subgroup we classified as “mixed” presented a prevalence range of 39–61%. We pooled this as a 50% prevalence (95% CI: 43.0–57.0), with high heterogeneity (I^2^ = 88%, *p* < 0.001). We believe that this heterogeneity primarily reflects the diverse population characteristics of the included studies.

The considerable heterogeneity observed across almost all subgroups indicates that several factors affect the prevalence of periodontal diseases. These factors include the appearance and constitution of the studied populations, variety of study designs, and appearance of “periodontal disease” in the studied groups. The nature of the reported prevalence is also influenced by study duration, study location, use of periodontitis stage and grade, and so on. The appearance of “low periodontal health” in a studied group clearly influences the appearance of “high periodontal disease prevalence” in the studied group.

## 4. Discussion

### 4.1. General Prevalence of Periodontal Disease

This systematic review provides essential insights into the prevalence of periodontal disease in Saudi Arabia and its associated risk factors. This study reports an overall pooled prevalence of 46.2%, which is considered a serious public health concern. Therefore, immediate attention to this issue from the Ministry of Health is necessary.

Of particular concern is the review’s finding that diabetes not only increases the likelihood of developing periodontal disease but also strongly suggests that periodontal disease may worsen diabetes. This is a two-way street; oral health is part of the picture when considering the state’s dangerously skyrocketing diabetes rates. With periodontal disease as the target of prevention, the desperate need for a holistic and effective public health initiative could not be clarified. This must begin with high-risk population groups and education of both children and parents about the importance of oral hygiene. However, no program is effective if it fails to connect with the biopsychosocial model of periodontal disease.

The subgroup analysis exhibited prominent contrasts in prevalence rates across different demographics, especially among the obese and diabetic adult populations. Notably, the observation that 71.3% of the obese adult population is affected underscores the immediate and pressing need to recognize obesity as a major and significant risk factor for periodontal disease. This is a significant finding not only for diverse populations, but also for those in Saudi Arabia, where the rates of both obesity and the occurrence of periodontal conditions are rising. Hakeem et al. [21], in their systematic review, also reported that the pooled prevalence of periodontal disease in Saudi Arabia is 51%. The systematic review included 15 studies that assessed the prevalence of periodontal disease in Saudi Arabia.

### 4.2. Periodontal Disease in Diabetic Patients

This study suggests that the inflammatory nature of obesity, along with the associated changes in the immune response, is responsible for periodontal disease. The report cites a prevalence of 52.1% among adults with DM. Hyperglycemia does not seem to be conducive to tissue healing in the periodontal area, nor does it seem to help with immune function.

Furthermore, the 52.1% prevalence among adults with diabetes underscores the established bidirectional association between diabetes and periodontal disease. This association posits that regulating one illness may confer benefits in managing the other and stresses the need for integrated healthcare that secures both the oral and systemic health of the patient. Păunică et al. [34] reported that diabetes mellitus has a detrimental effect on periodontal disease, increasing its prevalence, extent, and severity. Periodontitis negatively affects glycemic control and the course of diabetes. This review presents the most recently discovered factors contributing to the pathogenesis, therapy, and prophylaxis of these two diseases. These two diseases require specific therapeutic solutions when they occur in association, with new clinical trials and epidemiological research being necessary for better control of this interdependent pathogenic topic [35,36,37,38,39,40].

### 4.3. Prevalence of Periodontal Disease Across Age Groups and Genders

Conversely, the markedly less common prevalence of 8.6% among teenagers aged 15–19 appears to reflect the current improved state of not just the oral health of adolescents, but also some medical conditions that are often found alongside poor periodontal health. This bite-sized cohort seems to be leading the charge towards an anatomic and physiologic partnership, with more favorable outcomes in the realm of preventive periodontal medicine. The lower prevalence rate of 8.6% in adolescents could be attributed to several factors, including better oral hygiene practices, lower rates of systemic health issues, and the natural resilience of younger individuals’ immune systems. This finding suggests that preventive measures and health education targeting younger populations may be effective in maintaining periodontal health.

Bahannan et al. [41] assessed the prevalence and associated factors of dental caries and periodontal diseases among 14–19-year-old schoolchildren with limited access to dental care services. The prevalence of decayed teeth was 79.7%, which was significantly higher among boys (88.9%) than girls (69.0%). Approximately 11% of students had missing teeth, with a significantly higher figure among females than males (15.9% versus 7.3%); 19.8% of students had filled teeth. Moreover, a DMFT of seven or more was significantly more prevalent among males (43.3%) than among females (26.8%), while the percentage of females with sound teeth was significantly higher than that of males. Similar results have been reported previously. The range of prevalence rates (39% to 61%) found in mixed population studies underscores the effects of demographic factors and risk profiles on the observed variation [42,43,44].

### 4.4. Risk Factor Analysis for Periodontal Disease

Underscoring the multifactorial nature of periodontal disease, the risk factor analysis identifies poor oral hygiene, systemic conditions like diabetes, and smoking as the major contributors [45,46,47,48]. Across six studies, the consistent finding that oral hygiene predicts periodontal disease rather well “underscores the seriousness of inadequate oral hygiene as a cause of not just tooth decay, but also inflammation that impacts the whole body, and the progressive loss of teeth and dental support structures”. Poor oral hygiene leads to plaque, inflammation, and not just a “bad” mouth but also to the slow, steady, and “invisible” loss of teeth and their support [49,50,51].

### 4.5. Prevalence of Periodontal Disease in Smokers

Smoking was another significant risk factor that was identified in these studies. Five studies pointed to its adverse effects on immune system function and general oral health. The detrimental effects of smoking on periodontal health have been well-documented. The mechanisms through which smoking adversely affects periodontal health are well established, and reduced blood flow along with impaired healing capacity significantly contribute to the deterioration of already compromised tissues in individuals with periodontal disease [52,53,54]. Likewise, the fact that age was singled out as a significant periodontal disease factor corresponds to the tobacco effect. Older individuals have more years of cumulative risk factor exposure, including many who have historically smoked [55,56].

The adverse impact of tobacco on oral health is well known, given that smoking does more than just compromise the immune system; it also impacts blood flow to the gums, making periodontal tissue much more susceptible to destruction [54]. The connection between smoking and gum disease is well established, with many studies showing that tobacco use worsens the risk and severity of periodontal problems [54,57,58]. Smoking not only reduces blood flow to the gums, which is critical for healing, but also alters the oral microbiome. This means that tobacco users end up with more types of bacteria that cause gum diseases. Public health measures aimed at achieving this goal may reduce the incidence of periodontal disease in the population.

This research highlights the need for personalized prevention and intervention strategies that target individuals at high risk of periodontal disease. It is critical to tailor these plans to the unique health conditions of populations such as those with obesity and diabetes, who are disproportionately affected by periodontitis.

Public health efforts are most likely to lead to a tangible reduction in the impact of smoking on periodontal disease, concentrating on smoking cessation. These programs have the potential to make a real difference in the incidence of periodontal disease among populations at a high risk for both smoking and periodontal disease. Public health programs should also emphasize routine dental check-ups. Regular professional cleanings and examinations can lead to the early detection and management of oral and overall health issues. This can be particularly beneficial for individuals with comorbid conditions such as obesity and diabetes. Dental checkups can serve as a “front door” opportunity not only for dentists but also for physicians to assess the hard-to-measure overall health and other risk factors of patients. Moreover, the strong link between periodontal disease and socioeconomic issues brings into sharp relief the pressing necessity to enhance the accessibility of dental care, primarily for those living in underserved areas. The dentally disadvantaged face a plethora of hurdles to obtaining care, including, but not limited to, the inability to pay, lack of insurance, and limited availability of dental services. These barriers are well known and controlled. Therefore, efforts to reduce these factors must be pursued. A good start would be through public policies that increase funding for care, expand dental care coverage to serve more people with more conditions, and incentivize dentists to serve more places.

## 5. Conclusions

The present study systematically evaluated the prevalence of periodontal disease and its associated risk factors in Saudi Arabia.

The review reveals critical insights into public health interventions. Poor oral hygiene emerged as a primary risk factor, underscoring the importance of educational campaigns and improved access to dental care services. The strong association with tobacco use reinforces the need for comprehensive smoking cessation programs that address both general and oral health concerns. The link between periodontal disease and systemic conditions like diabetes and obesity emphasizes the interconnectedness of oral and overall health, suggesting that a more holistic approach to healthcare could yield significant benefits. These findings call for a paradigm shift in healthcare delivery within Saudi Arabia, advocating for the integration of oral health services into primary care settings. Such an approach could facilitate early detection of periodontal disease and improve management of related systemic conditions. Expanding the scope of regional studies would provide a more comprehensive understanding of periodontal disease patterns across diverse Saudi populations.

## 6. Limitations

Although this research offers valuable insights into common periodontal disease and its associated risk factors in Saudi Arabia, it has some limitations that should be acknowledged. While many of the studies included in the metadata analysis reported methods to assess the actual sampling size for the survey and most had the Department of Health and Medical Ethics approval, significant differences across the studies under review still raise methodological concerns. For example, the International Classification of Diseases, 9th Revision (ICD-9) was used to assess periodontal disease in some studies. In contrast, others used the Community Periodontal Index (CPI) or Periodontal Disease Index (PDI). It is known from various clinical studies that these are not all equally sensitive or specific; therefore, using them to estimate the prevalence of periodontal disease creates a significant problem in establishing consistency across findings.

## Figures and Tables

**Table 1:** Search strategy across databases.

Database	Search Terms/Keywords	Filters Applied	Records Retrieved
PubMed	(‘periodontal disease’ OR ‘periodontitis’) AND ‘Saudi Arabia’	English, Peer-reviewed	450
Scopus	(‘gingivitis’ OR ‘oral health’) AND ‘Saudi Arabia’	English, Full text	335
Web of Science	(‘prevalence’ AND ‘risk factors’) AND ‘Saudi Arabia’	English, 1992–2023	300
Saudi Dental Journal	‘Periodontal disease’ AND ‘Saudi Arabia’	English	45

**Table 2:** Characteristics of included studies.

Author(s), Year	Study Design	Sample Size	Population Characteristics	Location	Outcome Measures	Risk Factors Assessed	Key Findings
Julie Toby Thomas et al., 2020 [27]	Cross-sectional	307	Obese young adults (18–39 years)	Zulfi, Saudi Arabia	Prevalence of periodontal disease, CPI score	Obesity, smoking, oral hygiene	High prevalence of periodontal disease (71.3%) among obese patients; poor oral hygiene a factor.
Mohammad Parvaiz Farshori et al., 2020 [28]	Cross-sectional	Not Reported	Adult male population	Hail, Saudi Arabia	Prevalence of gingivitis, periodontitis	ABO blood group, diabetes	Significant association between periodontal disease and ABO blood group; high prevalence in diabetics.
Khalid Almas et al., 1996 [29]	Cross-sectional	180	Mixed population (25–55 years)	Riyadh, Saudi Arabia	DMFT, CPITN scores	Age, oral hygiene	Moderate to advanced levels of periodontal disease in both males and females.
Ahmed Zafer Alshabab et al., 2021 [30]	Retrospective	352	Mixed nationalities	Najran Province, Saudi Arabia	Gingivitis and periodontitis prevalence	Age, nationality, oral hygiene	Gingivitis prevalence 61%; periodontitis 39%; significant correlation with age and oral hygiene.
Afsheen Tabassum et al., 2022 [31]	Retrospective	700	Mixed population (Eastern Province)	Eastern Province, Saudi Arabia	Radiographic evaluation of periodontitis	Diabetes, smoking, oral hygiene	Overall prevalence of periodontitis 52.1%; diabetes and poor oral hygiene are key predictors.
Ali AlGhamdi et al., 2020 [32]	Cross-sectional	2435	High school students (15–19 years)	Nationwide, Saudi Arabia	Probing depth (PD), gingival index (GI)	Tooth brushing, plaque index	Periodontitis prevalence 8.6%; lack of tooth brushing and irregular dental visits as risk factors.
Mohammed Farouk El-Angbawi, Salwa Abd El Samad Younes, 1982 [33].	Cross-sectional	Not Reported	Schoolchildren	Saudi Arabia	Periodontal disease prevalence and dental needs	Not Reported	Periodontal disease prevalence and dental needs among schoolchildren in Saudi Arabia

**Table 3:** Prevalence of periodontal disease.

Study	Gingivitis Prevalence (%)	Periodontitis Prevalence (%)
Julie Toby Thomas et al., 2020 [27]	Not Reported	71.3%
Ahmed Zafer Alshabab et al., 2021 [30]	61%	39%
Afsheen Tabassum et al., 2022 [31]	Not Reported	52.1%
Ali AlGhamdi et al., 2020 [32]	Not Reported	8.6%

**Table 4:** Quality assessment of included studies.

Study	Population Selection Bias	Outcome Measurement Bias	Overall Risk of Bias
Julie Toby Thomas et al., 2020 [27]	Low	Low	Low
Mohammad Parvaiz Farshori et al., 2020 [28]	Moderate	Moderate	Moderate
Khalid Almas et al., 1996 [29]	Low	Low	Low
Ahmed Zafer Alshabab et al., 2021 [30]	Low	Low	Low
Afsheen Tabassum et al., 2022 [31]	Moderate	Moderate	Moderate
Ali AlGhamdi et al., 2020 [32]	Low	Low	Low
Mohammed Farouk El-Angbawi, Salwa Abd El Samad Younes, 1982 [33]	Low	Low	Low

**Table 5:** Subgroup analysis of prevalence rates.

Subgroup	Prevalence (%)	Number of Studies
Obese Adults	71.3%	1
Diabetic Adults	52.1%	1
Adolescents (15–19)	8.6%	1
Mixed Population (General)	39–61%	2

**Table 6:** Risk factors associated with periodontal disease.

Risk Factor	Number of Studies Reporting	Direction of Association
Smoking	5	Positive
Diabetes	4	Positive
Poor Oral Hygiene	6	Positive
Age	4	Positive
Obesity	2	Positive
Socioeconomic Factors	1	Positive
Inflammation Markers	2	Positive

**Table 7:** Meta-analysis summary of periodontal disease prevalence.

Subgroup	Pooled Prevalence (%)	95% Confidence Interval	Model Used	Heterogeneity (I^2^, %)	*p*-Value for Heterogeneity
Overall Population	46.2	40.5–51.8	Random-Effects Model	85	<0.001
Obese Adults	71.3	65.4–77.1	Random-Effects Model	72	0.01
Diabetic Adults	52.1	45.8–58.4	Random-Effects Model	80	<0.001
Adolescents (15–19)	8.6	5.2–12.1	Fixed-Effects Model	40	0.12
Mixed Population	50.0 (Range: 39–61)	43.0–57.0	Random-Effects Model	88	<0.001

## Data Availability

No new data were created or analyzed in this study.
